# Election of Delegates to National Association

**Published:** 1898-05

**Authors:** Emma Eames Chase

**Affiliations:** Corresponding Secretary National Dental Association


### ELECTION OF DELEGATES TO NATIONAL ASSOCIATION.

To the Editor:

Sir,—Will you please call attention to that section of the by-laws of the National Dental Association which relates to the appointment and qualifications of delegates, which is as follows:

"Article III., Section 3.—All delegate members shall be practitioners of dentistry. They shall be received only from permanently organized State dental societies. They shall be elected by ballot at some regular meeting of their society, and shall be members who have done meritorious work for the profession; but no person shall be received as a delegate who is in arrears for dues to this Association."

Also,

"Article IV., Section 1.—Each State society may send one for every ten of its active members, as delegates to this Association for one year, upon complying with the requirements of its constitution; but no society shall be entitled to representation that does not adopt or substantially recognize the Code of Ethics of this Association."

The fact that the *American* Dental Association received delegates from both local and State societies renders it necessary to call attention to the fact that delegates to the *National Dental Association* will be accepted only from the *State* societies, and that such delegates must be elected by ballot at a regular meeting of the society.

By request of the President.

EMMA EAMES CHASE,
*Corresponding Secretary National Dental Association.*

April 4, 1898.

[Will some one explain the meaning of Section 7, Article XIV., "Each branch may receive delegates from societies within its division, . . . *and such delegates shall have the same standing in the National Association as though admitted directly from the State societies.*" (Italics ours.)—ED.]

